# Machine learning algorithms for predicting and identifying the influencing predictors of antenatal care visits among women in Bangladesh: Evidence from BDHS 2022 data

**DOI:** 10.1371/journal.pone.0324226

**Published:** 2025-10-31

**Authors:** Md. A. Salam, Md. Merajul Islam, Md. Rezaul Karim

**Affiliations:** 1 Department of Statistics, University of Rajshahi, Rajshahi, Bangladesh; 2 Department of Statistics, Jatiya Kabi Kazi Nazrul Islam University, Mymensingh, Bangladesh; Dhaka University, BANGLADESH

## Abstract

**Background and Objective:**

Bangladesh, a South Asian country, continues to face significant challenges in maternal health, as reflected by its high maternal mortality ratio (MMR). According to the 2022 Bangladesh Demographic and Health Survey (BDHS), the MMR is 156 deaths per 100,000 births. This figure highlights ongoing challenges in maternal healthcare, despite improvements in recent years. Utilizing antenatal care (ANC) is a crucial intervention for reducing maternal mortality, as it enables early detection and treatment of complications, promotes health-seeking behavior, and prepares women for a safe childbirth. Thus, this study aimed to apply machine learning algorithms to predict the status of ANC visits and identify influential predictors among women in Bangladesh.

**Materials and Methods:**

The study used BDHS 2022 data of 5,128 women aged 15–49 years. The outcome variable was ANC, defined as having at least four visits during pregnancy. We employed Boruta and Stepwise regression to identify the important predictors associated with ANC. Subsequently, ten different machine learning algorithms— decision tree, random forest, artificial neural network, logistic regression, adaptive boosting, extreme gradient boosting, gradient boosting, k-nearest neighbors, ranger (RG), and support vector machine—were trained on the training set to predict ANC visits. The predictive performance of the models was evaluated using accuracy, precision, recall, F1-score, and AUC on the test set,

**Results,:**

The RG model performed best in predicting ANC visit status, with an accuracy of 69.46%, a precision of 68.51%, a Recall of 80.80%, an F1-score of 77.72%, and an AUC of 0.734, compared to the other models. The RG model identified age, wealth index, region, husband’s education, respondent education, and place of residence as the influential predictors of ANC utilization among women in Bangladesh,

**Conclusion:**

The RG model and the identified influential predictors offer valuable insights for designing targeted public health strategies to enhance ANC utilization among women in Bangladesh.

## 1. Introduction

Maternal morbidity and mortality pose significant challenges to global human development. Over 99% of maternal deaths occur in low- and middle-income countries, with Southern Asia (66%) and Sub-Saharan Africa (20%) accounting for approximately 86% of the global total [1]. The Millennium Development Goal (MDG) 5 aimed to reduce maternal deaths by 75% between 2000 and 2015 [2], while Sustainable Development Goal (SDG) 3.1 sets a target to lower the maternal mortality ratio (MMR) to below 70 per 100,000 live births by 2030 [3]. Most maternal deaths result from pregnancy-related complications, which are largely preventable through adequate antenatal care (ANC) and access to institutional delivery services. ANC plays a crucial role in reducing maternal and neonatal mortality by enabling routine check-ups that detect and manage conditions such as anaemia, hypertension, and infections early in pregnancy. It also allows healthcare providers to monitor fetal growth and development, facilitating timely interventions when abnormalities arise—thereby improving outcomes for both mother and child [4]. Moreover, ANC provides personalized guidance on healthy lifestyle practices, including proper nutrition, physical activity, and avoidance of harmful substances, empowering women to make informed decisions.

Despite the critical role of ANC, approximately 39% of pregnant women globally do not receive the recommended number of ANC visits [5]. In low- and middle-income countries, particularly in Asia and Africa, this figure rises to approximately 50%. In Bangladesh, around 53% of pregnant women fail to complete the recommended minimum of four ANC visits [6]. Inadequate utilization of ANC services is associated with significant economic and social costs. Although the availability of ANC services in public facilities, many women, particularly those from low-income and rural households, face hidden costs such as transportation, long waiting times, and lost income due to time away from work or household responsibilities. A study conducted by Jo et al. [7] found that rural women in Bangladesh often spend approximately 100 Bangladeshi Taka per visit on transport alone. When multiple visits are needed, this cost becomes a substantial burden, discouraging many women from completing the full ANC schedule. Consequently, women may delay or skip essential care, increasing risks for both maternal and neonatal health. By facilitating early detection of high-risk pregnancies, ANC enables healthcare systems to allocate targeted resources and specialized care, especially for vulnerable populations. This approach reduces health disparities and promotes equity in maternal health. However, disparities in ANC utilization persist. Only 47% of women in Bangladesh receive the recommended four or more ANC visits, and coverage is significantly lower among the poorest households compared to the wealthiest [8]. These economic and structural barriers reinforce existing inequalities and contribute to preventable maternal and neonatal morbidity and mortality. Therefore, ensuring adequate access to ANC is essential. Predicting ANC visits and identifying the key risk factors influencing utilization can support evidence-based interventions and policy decisions. Such predictive analysis is crucial for improving maternal health outcomes and promoting equity among pregnant women in Bangladesh.

Previous studies have identified several risk factors influencing ANC visits among women in Bangladesh [9–13]. These studies employed traditional statistical methods, such as logistic regression, to investigate the relationship between various factors and ANC utilization. While these methods are useful for understanding associations, they do not possess strong predictive capabilities. As a result, they may miss complex or non-linear patterns in the data that could help explain ANC visit behavior more effectively. This gap underscores the need for modern analytical approaches that incorporate predictive modeling to enhance understanding and improve ANC service utilization. Machine learning (ML), a subset of artificial intelligence (AI), has emerged as a powerful tool capable of automatically detecting hidden patterns and interactions within large datasets. ML algorithms have demonstrated strong performance in making accurate predictions and have increasingly been applied to classify ANC visit status [14]. These models can offer actionable insights, enabling healthcare professionals and policymakers to design more targeted and effective interventions to improve ANC uptake. Despite the growing global interest in ML-based approaches, there is a noticeable lack of studies applying these methods to recent Bangladeshi data. Specifically, no significant research has yet utilized the most recent Bangladesh Demographic and Health Survey (BDHS) 2022 dataset for this purpose. To address this gap, this study aims to implement several machine learning algorithms to develop a suitable predictive model for classifying ANC visit status and to identify the most influential factors associated with ANC utilization in Bangladesh. By incorporating nationally representative BDHS 2022 data, the findings from this study can contribute to the development of evidence-based strategies that enhance ANC service coverage and, ultimately, improve maternal and neonatal health outcomes in Bangladesh.

The remaining parts of the study are organized as follows. Section 2 outlines the materials and methods of the study. Section 3 presents the results of the data analysis. The discussion and conclusion of the study are presented in Sections 4 and 5, respectively.

## 2. Materials and methods

### 2.1 Study design and Data Source

This study employed a cross-sectional design using data from the BDHS, 2022. The BDHS serves as a nationally representative survey conducted on population, health, and nutrition indicators across Bangladesh. The survey followed a two-stage stratified sampling and includes information on women’s reproductive health, including ANC utilization. In the first stage, 675 enumeration areas (EAs) were selected using probability proportional to size, comprising 438 rural and 227 urban areas. In the second stage, a total of 30,375 residential households were surveyed, comprising 10,665 in urban areas and 19,710 in rural areas. It was anticipated that approximately 30340 interviews would be completed with ever-married women aged 15–49, including 10532 from urban areas and 19808 from rural areas. After eliminating missing and irrelevant observations, 5128 women were selected for this study.

#### 2.1.1. Outcome Variable.

The outcome variable in this study was ANC utilization, categorized into two groups based on the number of ANC visits received during a women’s pregnancy [15–18]. Women who received four or more ANC visits were coded as “Yes” (1), and those who received fewer than four or no visits were coded as “No” (0).

#### 2.1.2. Explanatory Variables.

Based on the findings from earlier studies on ANC visits and the availability of data on the relevant variables in the BDHS 2022 dataset were considered in this study [19–25]. The variables include mother age categorized in 5-year aged groups (15–19, 20–24, 25–29, 30–34, 35–39, 40–44, 45–49), geographical divisions (Barisal, Chittagong, Dhaka, Khulna, Mymensingh, Rajshahi, Rangpur, Sylhet), types of residenc (Urban, Rural), educational level of the respondents (No education, Primary, Secondary, Higher), sex of the household head (Male, Female), wealth index status (Poorest, Poorer, Middle, Richer, Richest), educational level of the respondent’s husband (No education, Primary, Secondary, Higher), working status of the respondent (Yes, No), health insurance status (Yes, No), number of household members (≤4, > 4), and total number of children in the household (≤3, > 3).

**Ethical approval:** As the data is available to the public on their website, it is not required to obtain ethical review and approval for this research involving human participants in compliance with local laws and institutional regulations.

### 2.2. Feature Selection

In machine learning, feature selection involves identifying the most relevant features from a larger set to improve model performance. Focusing on the most informative features helps reduce computational complexity and minimizes the risk of overfitting. Feature selection also enhances the interpretability of the model, providing better insights into the data’s underlying patterns. This study employed Boruta and stepwise logistic regression methods to select the most important features of ANC.

#### 2.2.1 Boruta.

The Boruta is a widely used wrapper-based feature selection method that enhances the performance of ML-based models by identifying important features from the dataset using the random forest (RF) classifier [26]. It works by creating shadow features, which are duplicates of the original features with their values randomly shuffled. These shadow features serve as a reference to assess the importance of the actual features. Boruta then trains the RF model on the combined dataset of original and shadow features and computes the importance score for each feature. It compares the importance score of each original feature with the highest importance score among the shadow features. If a feature consistently performs better than the shadow features, it is marked as important. If it performs worse, it is deemed unimportant, and if the result is inconclusive, it is considered tentative. This process is repeated until all features are clearly classified as either important or unimportant, or until a predefined number of iterations has been reached.

#### 2.2.2. Stepwise Logistic Regression.

To identify the important predictors of ANC visit utilization, we employed the stepwise binary logistic regression method [27]. This method combines forward selection and backward elimination procedures to automatically select variables based on their statistical significance in contributing to the model. The probability that a binary outcome variable Y=1 (ANC visits) given a set of input variables $X_{\text{1}},X_{\text{2}},...,X_{k}$. The model is expressed as:


$$logit(p)=ln\left(\frac{p}{1-p}\right)=\beta_{\text{0}}+\beta_{\text{1}}X_{\text{1}}+\beta_{\text{2}}X_{\text{2}}+\ldots +\beta_{k}X_{k}$$

p is the probability that Y=1, $\beta_{\text{0}}$ is the intercept,$\beta_{\text{1}},\beta_{\text{2}},...,\beta_{k}$ are the coefficients for $X_{\text{1}},X_{\text{2}},...,X_{k}$, respectively. The odds ratio (OR) for a given input variable is calculated by exponentiating its coefficient:


$$OR=e^{\beta }$$

An OR > 1 indicates that the predictor increases the odds of ANC visits, while an OR < 1 indicates decreased odds. Each OR is accompanied by a 95% confidence interval (CI), calculated as:


$$CI=\left(e^{\beta -1.96\cdot SE},\;e^{\beta +1.96\cdot SE}\right)$$

A predictor with p-value <0.05 was considered statistically significant and identified as a risk factor associated with ANC visit utilization. After identifying the important predictors, the most important were determined using the following formula:


$$Most\;important\;predictors={⋂}_{i}^{r}{Predictors\;identification\;methods}_{i}\mathrm{\backslash ]}$$

here, r=2.

### 2.3 Machine Learning Algorithms

This study applied ten widely used machine learning algorithms, namely decision tree (DT), RF, artificial neural network (ANN), logistic regression (LR), adaptive boosting (AdaB), extreme gradient boosting (XGB), gradient boosting (GB), k-nearest neighbors (KNN), ranger (RG), and support vector machine (SVM). These algorithms have been selected due to their effectiveness in the classification tasks, ability to capture complex patterns in the data, and widespread use in similar studies, such as ANC [28–35]. The selected algorithms represent a broad range of modeling techniques, including tree-based (DT, AdaB, RF, GB, XGB, and RG), linear (LR), nonlinear (ANN and SVM), and instance-based (KNN). This diversity enabled a comprehensive performance across different algorithmic approaches, thereby enhancing the robustness of our findings.

#### 2.3.1. Decision Tree.

The decision tree (DT) is a tree-like structure, a non-parametric machine learning algorithm [36]. The tree begins with a root node, where a feature is evaluated, and branches out through decision nodes based on the feature values. This process continues until it reaches a leaf node, which represents the final output (class label or prediction).

#### 2.3.2. Random Forest.

Random forest (RF) is an ensemble-based learning algorithm used for both classification and regression. It builds multiple decision trees by creating bootstrap samples from the original data. At each split within a tree, a random subset of features is considered. For classification, the algorithm uses Gini impurity to decide the best splits. After all trees are constructed, RF combines their predictions by the majority voting, resulting in a more accurate and robust final prediction compared to a single DT [37].

#### 2.3.3. Artificial Neural Network.

Artificial neural network (ANN) is a computational model inspired by the structure and function of the human brain. It consists of an input layer, one or more hidden layers, and an output layer. Each connection between neurons has a weight that adjusts during training to minimize the prediction error [38]. The input data passes through these layers, where weighted sums and activation functions are applied to learn patterns and make predictions.

#### 2.3.4. Logistic Regression.

Logistic regression (LR) is a machine learning algorithm for binary classification that predicts the probability of an outcome by modeling the log-odds as a linear combination of the input predictors [39]. By applying the logistic (sigmoid) function, this log-odds is converted to a probability:


$$p=\frac{\text{1}}{1+e^{-(\beta_{\text{0}}+\beta_{\text{1}}x_{\text{1}}+\beta_{\text{2}}x_{\text{2}}+\cdots +\beta_{k}x_{k})}}$$

The model predicts the probability of the positive class for new data points and classifies them using the following decision rule:


$$\begin{array}{c} \hat{y}=\left\{ \begin{array}{c} 1\;if\;p\geq 0.5\\ 0\;if\;p<0.5 \end{array}\right.\; \end{array}$$

#### 2.3.5. Adaptive boosting.

Adaptive boosting (AdaB), an ensemble ML method similar to RF in that it combines multiple models to improve overall performance. However, unlike RF, which builds many independent trees, AdaB trains a sequence of weak learners—typically shallow DT—where each learner focuses more on the instances misclassified by the previous ones. During training, the algorithm assigns higher weights to the misclassified observations, encouraging subsequent models to pay more attention to them. The final prediction is made by combining the predictions of all learners using a weighted majority voting method, where more accurate learners have a greater influence on the final output [40].

#### 2.3.6. Gradient boosting.

Gradient boosting (GB), introduced by Jerome Friedman, is a powerful ensemble learning algorithm that build models sequentially to improve prediction perfomance. Unlike methods that train models independently, GB adds one tree at a time, where each new tree attempts to correct the errors made by the previous ones. It focuses more on the observations that earlier models poorly predicted. Although small and simple, these trees collectively form a strong predictive model. The final classification is typically made by aggregating the outputs of all trees, often through a weighted majority voting of predictions [41].

#### 2.3.7. Extreme gradient boosting.

Extreme gradient boosting (XGB) is an advanced implementation of the GB algorithm that uses DT as a base learner. Unlike RF, where trees are built independently and in parallel, XGB constructs trees sequentially, with each new tree aiming to correct the errors of the previous ones. This iterative process enhances prediction accuracy by focusing on the most challenging cases. XGB also allows fine control over model complexity, such as tree depth and learning rate, making it highly effective for building fast, scalable, and accurate models [42].

#### 2.3.8. K-Nearest Neighbor.

The k-nearest neighbors (KNN) algorithm is a simple classification method that assigns labels based on the majority class of the k closest data points. However, it suffers from low efficiency in large-scale tasks due to its lazy learning nature and is highly sensitive to the choice of k, which can significantly impact the model’s performance [43].

#### 2.3.9. Ranger.

Ranger (RG) is an efficient implementation of the RF algorithm, optimized for speed and capable of handling large datasets. In the first stage, three short-term load forecasting (STLF) models are developed using tree-based ensemble learning techniques on the training set. In the second stage, a ranger-based prediction model is implemented to enhance prediction performance by incorporating the strengths of RF in handling complex, high-dimensional data and capturing nonlinear relationships [39].

#### 2.3.10. Support vector machine.

Support Vector Machine (SVM) is a powerful supervised machine learning algorithm used for classification and regression tasks. It works by identifying the optimal hyperplane that maximally separates data points of different classes in a high-dimensional space. SVM performs well in complex and high-dimensional data by maximizing the margin between classes. To handle nonlinear relationships, it uses kernel functions. In this study, the radial basis function (RBF) kernel was employed to capture nonlinear patterns and improve classification performance [44].

### 2.4. Cross validation and hyperparameter tuning

K-fold cross-validation (CV) is a widely used protocol in machine learning that provides more comprehensive assessment of a model’s performance than a single train-test split [40]. In K-fold, the original dataset is divided into K equal-sized subsets or folds. The process is repeated K times, where each time, one of the K subsets is used as the validation or test set, while the remaining K-1 subsets are combined to form the training set. The final model performance is determined by averaging the accuracy across all subsets. In this study, K was set to 10. The hyperparamers of these models were tuned using the grid search method to optimize model performance.

### 2.5. Model evaluation

Model evaluation measures the performance of a model to assess its effectiveness using various metrics on the test set [45]. The confusion matrix summarizes how well a classification model predicts actual versus predicted classes [Table 1]. It includes true positives, true negatives, false positives, and false negatives, which are used to calculate the evaluation metrics: accuracy, precision, recall, and F1-score.

**Table 1:** Confusion matrix.

		Predictive Class	
		Yes	No
Actual Class	Yes	True Positive (TP)	False Negative (FN)
	No	False Positive (FP)	True Negative (TN)

**Accuracy** Accuracy measures the number of correctly predicted instances as a percentage of the total instances. Its mathematical expression is as follows:


$$Accuracy=\frac{TP+TN}{TP+TN+FN+FP}$$

**Precision** Precision measures the correctly predicted positive instances out of all instances predicted as positive. Its mathematical expression is as follows:


$$Precision=\frac{TP}{TP+FP}$$

**Recall** Recall measures the actual positive instances correctly identified by the model. Its mathematical expression is as follows:


$$Recall=\frac{TP}{TP+FN}$$

**F1-score** The F1-score is the harmonic mean of Precision and Recall, providing a balanced measure that combines both metrics. Its mathematical expression is as follows:


$$F1-score=\frac{2\times Recall\times Precision}{Recall+Precision}$$

#### 2.5.1. ROC Curve.

**Fig 1:**
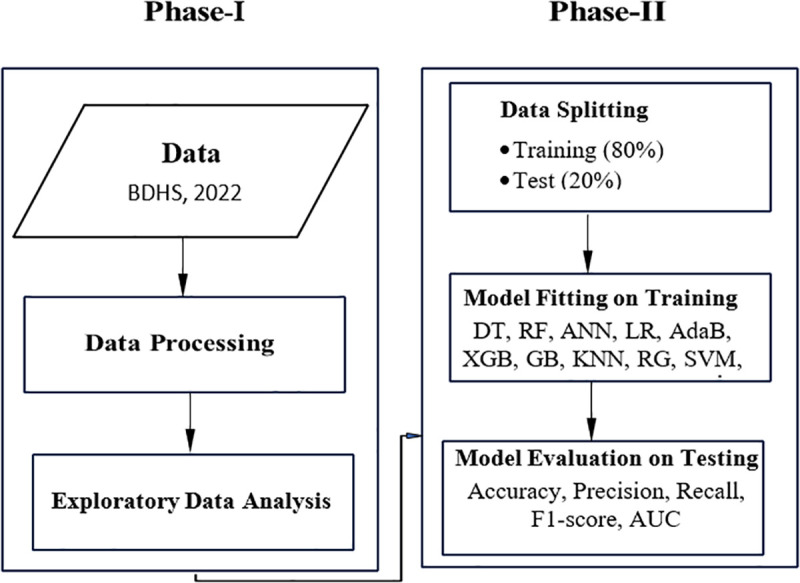
Flowchart illustrates the process for predicting ANC visits.

The receiver operating characteristic (ROC) curve is a visual representation used to evaluate the performance of binary classification models [46]. It illustrates how well a model distinguishes between true positive and false positive cases at different threshold values. The area under the ROC curve (AUC) measures the overall ability of the model to discriminate between classes. Finally, we identified the influential predictors of ANC visits for the best-performing model. The flowchart illustrating the process for predicting ANC visit status is shown in **Fig 1**.

## 3. Results

### 3.1 Socio-demographic characteristics of the study population

#### 3.1.1. Descriptive statistics.

A total of 5,128 women were included in the study to assess the status and predictors of ANC visits in Bangladesh. The majority of respondents were aged 20–29 years, with 32.3% in the 20–24 aged group and 28.2% in the 25–29 aged group. Most women resided in rural areas (67.1%) and had secondary education (52.7%), while 19.0% had higher education and 5.3% had no formal education. Approximately 88.9% of households were headed by males. Regarding wealth status, 20.8% belonged to the poorest group, and 19.4% were in the richest group. About 78.5% of women were not engaged in paid work, and nearly all (99.7%) lacked health insurance coverage. Most households had more than four members (88.6%), and 92.6% of the women had ≤3.

#### 3.1.2. ANC visits and their association with explanatory variables.

**Table 2** shows the relationship between explanatory variables and ANC visit utilization. The overall prevalence of pregnant women involved in ANC visits is 40.7%. The highest prevalence of ANC was observed among women aged 30–34 (44.3%), followed by those aged 25–29 (42.3%), 35–39 (40.7%), and 35.4% of those aged 15–19. The Mymensingh (47.1%), Dhaka (45.8%), and Khulna (45.2%) divisions showed higher ANC utilization, while the Sylhet (32.9%) and Barisal (34.4%) divisions had the lowest. Urban women had greater ANC utilization (54.1%) compared to their rural counterparts (34.1%). Only 20.7% of women with no education received ANC, compared to 26.8% with primary, 40.2% with secondary, and 64.4% with higher education. ANC utilization also increased with husbands’ education, ranging from 27.2% among women whose husbands had no education to 64.5% among those whose husbands attained higher education. Women from wealthier households had the highest ANC attendance at 66.5%, while those from the poorest socioeconomic group had the lowest attendance at 22.9%. Women covered by health insurance reported higher ANC coverage (64.7%) than those without coverage (40.6%). Moreover, household size showed a slight difference, with 45.1% of women from households of four or fewer members receiving ANC compared to 40.1% from larger households. Finally, ANC utilization was higher among women with three or fewer children (41.8%) than among those with more than three children (27.7%).

**Table 2:** Association between the expalanatory variables and ANC visits of pregnant women.

Explanatory variables	Overall n (%)	ANC visits status		p-value
		Yes, n (%)	No, n (%)	
**Age**				
15-19	687(13.4)	243(35.4)	444(64.6)	0.014
20-24	1658(32.3)	656(39.6)	1002(60.4)	
25-29	1446(28.2)	611(42.3)	835(57.7)	
30-34	888(17.3)	393(44.3)	495(55.7)	
35-39	371(7.2)	151(40.7)	220(59.3)	
40-44	70(1.4)	30(42.9)	40(57.1)	
45-49	8(0.2)	2(25.0)	6(75.0)	
**Region**				
Barisal	553(10.8)	190(34.4)	363(65.6)	<0.001
Chittagong	883(17.2)	348(39.4)	535(60.6)	
Dhaka	758(14.8)	347 (45.8)	411(54.2)	
Khulna	582(11.3)	263(45.2)	319(54.8)	
Mymensingh	633(12.3)	298(47.1)	335(52.9)	
Rajshahi	520(10.1)	213(41.0)	307(59.0)	
Rangpur	600(11.7)	230(38.3)	307(61.7)	
Sylhet	599(11.8)	197(32.9)	402(67.1)	
**Residence**				
Urban	1689(32.9)	914(54.1)	775(45.9)	<0.001
Rural	3439(67.1)	1172(34.1)	2267(65.9)	
**Education**				
None	270(5.3)	56(20.7)	214(79.3)	<0.001
Primary	1183(23.1)	317(26.8)	866(73.2)	
Secondary	2701(52.7)	1086(40.2)	1615(59.8)	
Higher	974(19.0)	627(64.4)	347(35.6)	
**Sex of household head**				
Male	4559(88.9)	1841(40.4)	2718(59.6)	0.220
Female	569(11.1)	245(43.1)	324(56.9)	
**Wealth index**				
Poorest	1067(20.8)	244(22.9)	823(77.1)	<0.001
Poorer	1034(20.2)	313(30.3)	721(69.7)	
Middle	1019(19.9)	379(37.2)	640(62.8)	
Richer	1011(19.7)	487(48.2)	524(51.8)	
Richest	997(19.4)	663(66.5)	334(33.5)	
**Husband education**				
None	765(14.9)	208(27.2)	557(72.8)	<0.001
Primary	1529(29.8)	448(29.3)	1081(70.7)	
Secondary	1775(34.6)	747(42.1)	1028(57.9)	
Higher	1059(20.7)	683(64.5)	376(35.5)	
**Working status**				
No	4027(78.5)	1658(41.2)	2369(58.8)	0.169
Yes	1101(21.5)	428(38.9)	673(61.1)	
**Health insurance**				
No	5111(99.7)	2075(40.6)	3036(59.4)	0.043
Yes	17(0.3)	11(64.7)	6(35.3)	
**Number of household members**				
** ≤ 4**	585(11.4)	264(45.1)	321(54.9)	0.02
** > 4**	4543(88.6)	1822 (40.1)	2721(59.9)	
**Total children**				
** ≤ 3**	4677(92.6)	1954(41.8)	2723(58.2)	<0.001
** > 3**	375(7.4)	104(27.7)	271(72.3)	

### 3.2. Feature Selection and Identification of Important Predictors

The important predictors for ANC visits were identified using two feature selection methods: Boruta and stepwise logistic regression.

#### 3.2.1. Risk Factors Identification using Boruta.

**Fig 2:**
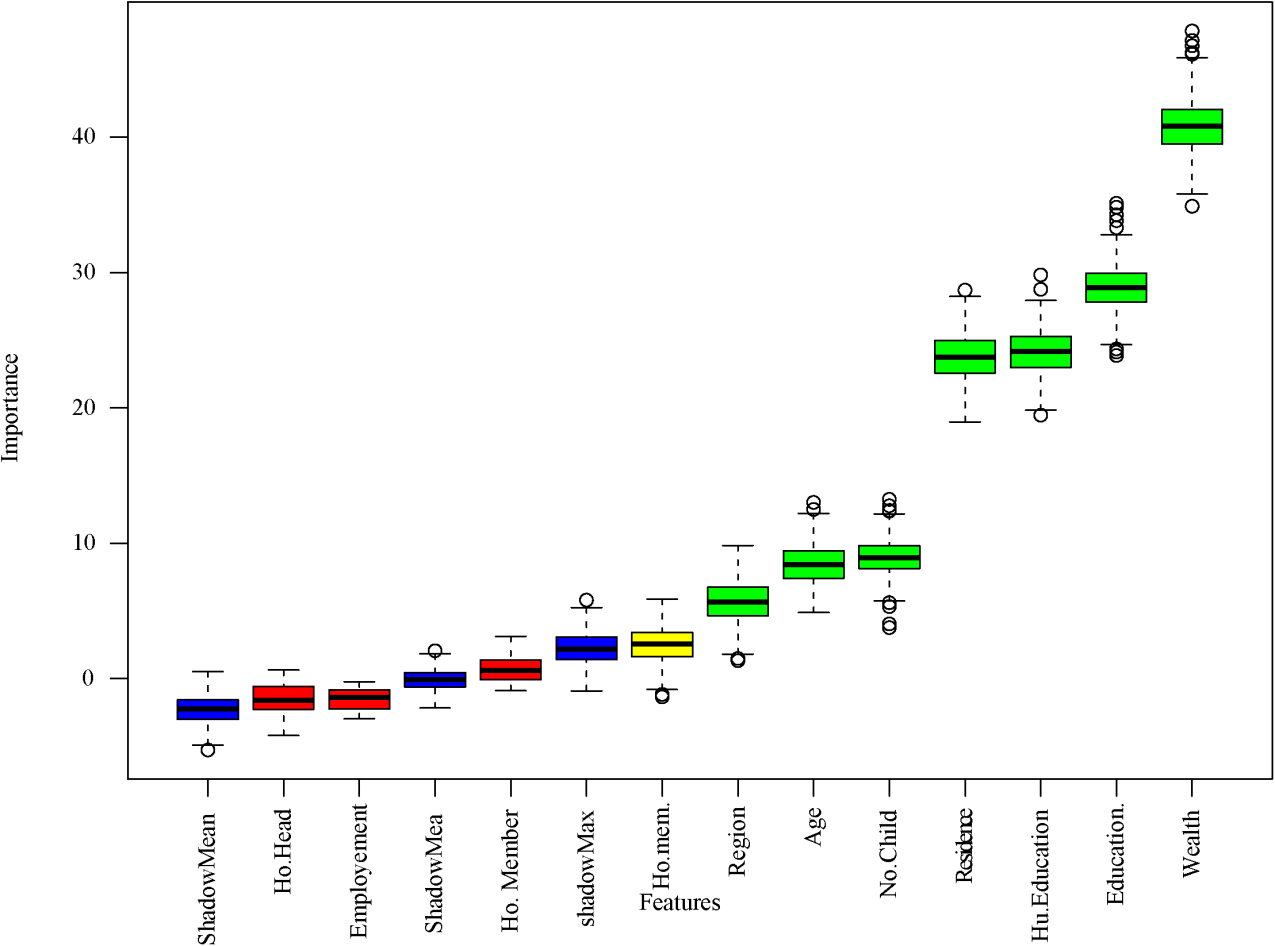
Identified important predictors for ANC visits by Boruta algorithm.

**Fig 2** illustrates the identification of important predictors using the Boruta feature selection method. In this figure, blue bars indicate significant predictors, while red bars represent those deemed insignificant. The Boruta algorithm identified the features as important for the wealth index, education, husband’s education, type of residence, number of children, age, and region.

#### 3.2.2. Risk factors identification using stepwise logistic regression.

The results of the LR method are presented in Table 3. It was observed that women with aged 15–19 years (OR: 0.700; 95% CI: 0.558–0.879; p = 0.002) and 20–24 years (OR: 0.770; 95% CI: 0.639–0.927; p = 0.006) had lower odds of attending ANC visits compared to women aged 30–34 years. Compared to women living in the Mymensingh division, those residing in Barisal (OR: 0.417; 95% CI: 0.322–0.540; p < 0.001), Chittagong (OR: 0.539; 95% CI: 0.429–0.676; p < 0.001), Dhaka (OR: 0.549; 95% CI: 0.432–0.697; p < 0.001), Khulna (OR: 0.568; 95% CI: 0.443–0.729; p < 0.001), Rajshahi (OR: 0.498; 95% CI: 0.384–0.645; p < 0.001), Rangpur (OR: 0.597; 95% CI: 0.466–0.763; p < 0.001), and Sylhet (OR: 0.449; 95% CI: 0.348–0.579; p < 0.001) had significantly lower odds of attending ANC visits. Women living in rural areas had significantly lower odds of receiving ANC visits compared to those women living in urban areas (OR: 0.627; 95% CI: 0.547–0.719; p < 0.001). Women with no education (OR: 0.353; 95% CI: 0.242–0.514; p < 0.001), primary education (OR: 0.466; 95% CI: 0.369–0.588; p < 0.001), and secondary education (OR: 0.696; 95% CI: 0.578–0.839; p < 0.001) had significantly lower odds of attending ANC visits compared to those with higher education. Women living in the poorest (OR: 0.323; 95% CI: 0.254–0.410; p < 0.001), poorer (OR: 0.411; 95% CI: 0.331–0.511; p < 0.001), middle (OR: 0.485; 95% CI: 0.396–0.594; p < 0.001), and richer (OR: 0.662; 95% CI: 0.545–0.804; p < 0.001) wealth groups were less likely to experience of the ANC visits compared to the richest group. Women whose husbands had no education (OR: 0.547; 95% CI: 0.424–0.706; p < 0.001), primary education (OR: 0.536; 95% CI: 0.434–0.661; p < 0.001), secondary education (OR: 0.657; 95% CI: 0.546–0.791; p < 0.001) had significantly lower odds of the ANC visits compared to those whose husbands had higher education. Women from households with ≥4 members had lower odds of attending ANC visits compared to those with < 4 (OR: 0.769; 95% CI: 0.635–0.931; p = 0.007).

**Table 3:** Identification of predictors of ANC visits using logistic regression.

Explanatory variables	Categories	OR (95% CI)	p-value
Age	30-34^*^		
	15-19	0.700(0.558-0.879)	0.002
	20-24	0.770(0.639-0.927)	0.006
	25-29	0.882(0.730-1.065)	0.191
	35-39	0.990(0.750-1.308)	0.945
	40-44	1.179(0.675-2.060)	0.562
	45-49	0.918(0.163-5.174)	0.923
Region	Mymensingh^*^		
	Barisal	0.417(0.322-0.540)	<0.001
	Chittagong	0.539(0.429-0.676)	<0.001
	Dhaka	0.549(0.432-0.697)	<0.001
	Khulna	0.568(0.443-0.729)	<0.001
	Rajshahi	0.498(0.384-0.645)	<0.001
	Rangpur	0.597(0.466-0.763)	<0.001
	Sylhet	0.449(0.348-0.579)	<0.001
Residence	Urban^*^		
	Rural	0.627(0.547-0.719)	<0.001
Education	Higher^*^		
	None	0.353(0.242-0.514)	<0.001
	Primary	0.466(0.369-0.588)	<0.001
	Secondary	0.696(0.578-0.839)	<0.001
Wealth index	Richest^*^		
	Poorest	0.323(0.254-0.410)	<0.001
	Poorer	0.411(0.331-0.511)	<0.001
	Middle	0.485(0.396-0.594)	<0.001
	Richer	0.662(0.545-0.804)	<0.001
Husband education	Higher^*^		
	None	0.547(0.424-0.706)	<0.001
	Primary	0.536(0.434-0.661)	<0.001
	Secondary	0.657(0.546-0.791)	<0.001
Health insurance	Yes^*^		
	No	0.500(0.169-1.475)	0.209
	≤4^*^		
	>4	0.769(0.635-0.931)	0.007
Number of living children	≤3^*^		
	>3	0.773(0.584-1.022)	0.071

Here, ‘*’ indicates the reference category.

The overlapping predictors identified by both the Boruta and LR methods include age, region, residence, education, wealth index, and husband’s education. These variables are considered the most important predictors of ANC visits and were incorporated into machine learning algorithms to develop models for predicting ANC visit status among women in Bangladesh.

### 3.3. Machine learning model performance and prediction

#### 3.3.1. Confusion matrix analysis.

**Fig 3:**
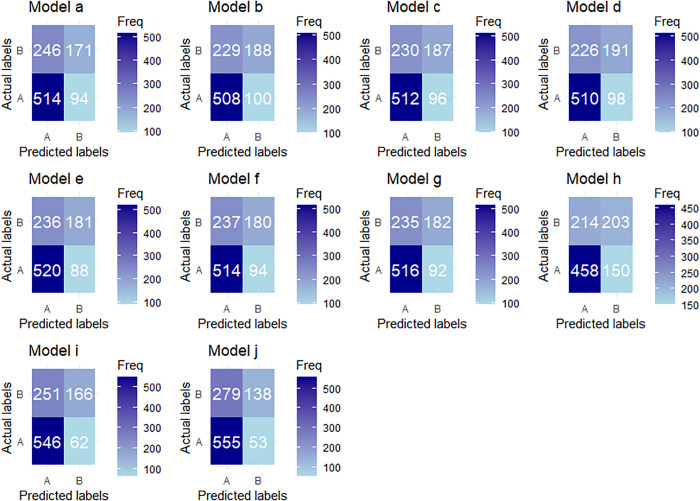
Confusion matrix for (a) DT, (b) RF, (c) ANN, (d) LR, (e) AdaBoost, (f) XGB, (g) GB, (h) KNN, (i) Ranger, and (j) SVM. Here, factor labels A and B represent 0 and 1, respectively.

To evaluate and compare the performance of the developed machine learning models, confusion matrices were generated for each classifier in the first phase, as shown in **Fig 3**. These matrices help show how many predictions were correct or incorrect for each model. In addition, several evaluation metrics—such as accuracy, precision, recall, F1-score, and the ROC curve with AUC—were calculated to assess model performance. To determine the best model, these metrics are considered simultaneously.

#### 3.3.2. Evaluation of model performance metrics.

The prediction performance of the machine learning models is shown in Table 4, reveals that RG is the best-performing model, achieving the highest accuracy of 69.46%. It demonstrated an excellent recall of 89.80%, indicating that it was highly effective in identifying actual positive cases. Notably, the model achieved the highest F1-score of 77.72%, indicating a strong balance between precision and recall. Although its precision (68.51%) is slightly lower than that of some other models, this trade-off is acceptable given its superior recall and overall balanced performance.

**Table 4:** Comparisons of the prediction performance of different models.

Model	Accuracy (%)	Precision (%)	Recall (%)	F1-score (%)
**DT**	66.83	67.63	84.54	75.15
**RF**	67.71	68.79	83.39	75.54
**ANN**	68.20	69.00	84.21	74.66
**LR**	68.39	69.29	83.88	75.89
**AdaB**	68.39	68.78	85.53	76.25
**XGB**	67.71	68.44	84.54	75.64
**GB**	68.10	68.71	84.87	75.94
**KNN**	64.49	68.15	75.33	71.56
**RG**	**69.46**	68.51	89.80	**77.72**
**SVM**	67.61	66.55	91.28	76.69

#### 3.3.2. Receiver operating characteristic (ROC) Curve Analysis.

**Fig 4:**
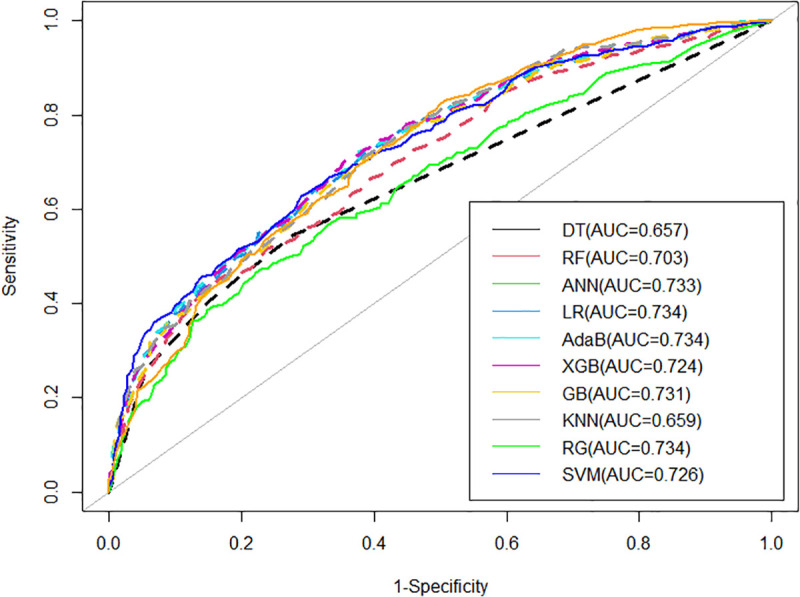
ROC curves of different ML-based models.

The ROC curves with AUC values are shown in **Fig 4** to compare the diagnostic efficacy of different ML models. In the figure, different colors represent different models, while the diagonal line indicates the reference. As the AUC values for LR, AdaB, and RG are equally the highest, it is necessary to evaluate additional performance metrics, as presented in Table 4, to determine the most effective model. Table 4 indicates that the RG model outperforms the others. Therefore, we propose that the RG model is the most appropriate for predicting ANC visits among women in Bangladesh.

#### 3.3.4. Influential Predictors of Antenatal Care Visits.

**Fig 5:**
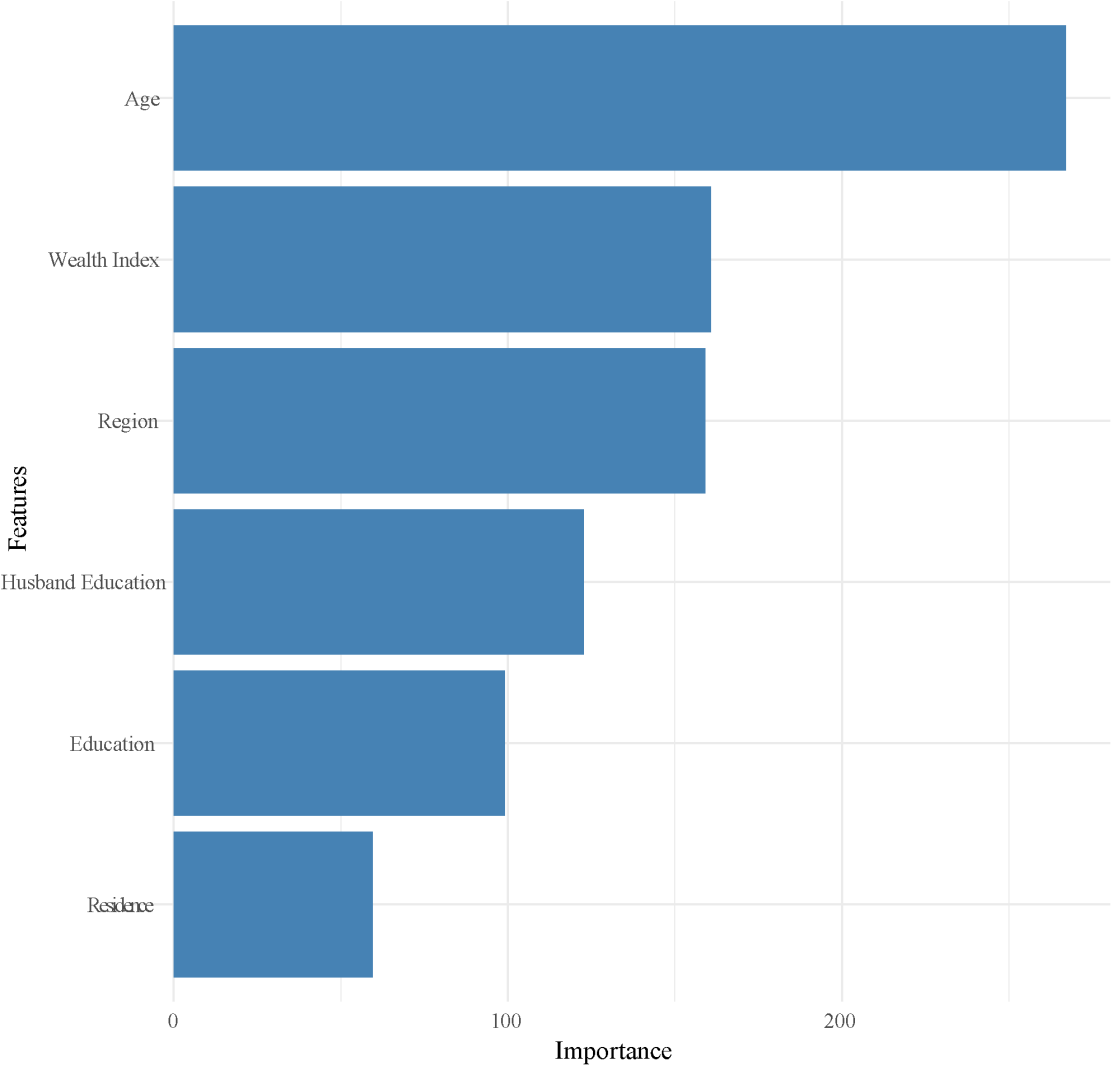
Influential predictors of ANC visits identified by the RG model.

The predictors of the best-performing RGmodel are presented in Fig 5. It was observed that age, wealth index, region, husband’s education, and place of residence are the influential predictors of ANC visits among women in Bangladesh.

## 4. Discussion

The prevalence of ANC visits in this study was 40.7%, which is notably lower than the findings from other countries such as India (51.6%), Ethiopia (62.8%), and Rwanda (54%) [47–49]. A lack of awareness, limited access to services, and inadequate understanding of service consumption among study participants may contribute to this. This study proposes a machine learning-based predictive model to predict ANC visits and identify key predictors influencing ANC utilization, thereby supporting targeted interventions to enhance maternal healthcare services. To achieve this objective, we utilized Boruta and stepwise logistic regression to identify the impotant predictors of ANC visits, using the most recent BDHS 2022 data. Both methods consistently identified the following predictors as important: mother’s age, household members, region of residence, wealth index, respondent’s education level, total number of children, type of place of residence, and husband’s education. Subsequently, we applied ten different machine learning algorithms, including DT, RF, ANN, LR, AdaB, XGB, GB, KNN, Ranger, and SVM, to predict ANC visits among women based on the identified predictors.

The performance of these models was evaluated using various metrics, including accuracy, precision, recall, F1-score, and AUC. These metrics provide a comprehensive assessment of each model’s ability to correctly predict ANC visits. Based on the evaluation of performance metrics, the RG model emerged as the best performer compared to the other models. After selecting the best model, the study then identified the influential predictors based on the RG model. It is to be observed that age, wealth index, region, husband education, education, and place of residence are the influential predictors of ANC. Age is associated with the utilization of ANC visits due to both physiological and social factors. Younger women often have higher risks for pregnancy complications, like preterm birth or low birth weight, which can lead to healthcare providers recommending more frequent ANC visits. However, younger women may face barriers, such as limited healthcare knowledge or lower decision-making power, making them less likely to attend ANC consistently. For older women, especially those above 35, age-related health risks like gestational diabetes, hypertension, and chromosomal abnormalities increase, prompting more ANC visits for monitoring and intervention [50–51]. Older women also tend to have greater awareness and financial stability, both of which support regular attendance at ANC. As a result, age shapes both the perceived need for ANC and the likelihood of accessing it, with younger and older women facing unique but impactful challenges and motivations. The wealth index is closely associated with the likelihood of ANC visits due to its influence on access to healthcare, resources, and awareness. Women from wealthier backgrounds often have the financial means to afford transportation, healthcare fees, and even private facilities, which can increase the frequency and quality of ANC visits. In contrast, women with lower wealth indices may struggle to cover these costs, which can reduce their ability to attend ANC regularly. Additionally, a higher wealth index often correlates with better education and health awareness, making wealthier women more likely to recognize the importance of ANC. Financial stability also enables women to take time off work or arrange childcare, reducing practical barriers to attending ANC appointments. Wealthier households are typically better connected to healthcare resources, which may further encourage consistent ANC. This financial capacity and awareness help bridge gaps in access to ANC, improving maternal and fetal health outcomes. Bangladesh is divided into eight divisions, each with varying levels of healthcare infrastructure and public health investment accessibility. Women living in more developed divisions (e.g., Dhaka or Khulna) often have better access to health services, more skilled health workers, and greater awareness of maternal healthcare. In contrast, women in underdeveloped or remote divisions (e.g., Barisal or Sylhet) may face challenges such as limited access to health facilities, long travel distances, cultural barriers, and lower exposure to health education campaigns. These regional disparities contribute to differences in ANC utilization across divisions. Husband’s education is associated with ANC visits because it often influences a family’s health decisions, resources, and support for maternal care.

An educated husband is more likely to understand the importance of utilization of ANC for the mother and baby’s health, leading him to encourage and support regular visits. Education provides husbands with a greater awareness of the risks involved in pregnancy and the preventive benefits of ANC, fostering a supportive environment for attending these visits. The husband’s education can also improve financial stability, allowing the family to afford transportation, healthcare costs, and time off work, all of which make ANC more accessible. Furthermore, educated husbands may be more inclined to prioritize maternal healthcare within the family budget and be more proactive in facilitating access to healthcare. In societies where husbands play a key role in decision-making, an educated husband is often more understanding of the necessity of ANC, reducing cultural or traditional barriers. His support thus becomes a critical factor in ensuring the mother receives consistent ANC, leading to better health outcomes for both mother and child. Women’s education is also vital in influencing ANC utilization due to its strong connection with health awareness and understanding. Educated women understand the importance of ANC for the health of both mother and baby. They have better access to information about pregnancy and ANC benefits, encouraging regular visits. Education fosters confidence, enabling women to make informed health decisions, including prioritizing ANC. Financial independence, often linked with education, helps women afford and access healthcare. Educated women also build wider social networks, where friends and family encourage them to visit ANC.

Higher health literacy from education makes it easier for women to follow medical advice. Education helps reduce cultural barriers, making women more receptive to modern healthcare. Overall, education promotes health-seeking behaviors, resulting in improved maternal and child health outcomes. Urban women are generally more likely to attend ANC services due to proximity to healthcare facilities, availability of skilled providers, and better transportation systems. They also tend to have higher education levels and greater exposure to mass media, which increases awareness about the importance of ANC. In contrast, women in rural areas often face physical, financial, and informational barriers. Long distances to health centers, poor road conditions, lower income levels, and traditional beliefs may hinder rural women’s access to timely and adequate ANC services. The predictors highlight key areas where policy interventions can enhance access to and outcomes of ANC. For age, policies should focus on tailored ANC programs for both younger and older pregnant women. This could include targeted outreach and education programs in schools and community centers for younger mothers to raise awareness about the importance of ANC. For older women, healthcare policies could provide specialized care options to monitor age-related risks such as hypertension or gestational diabetes.

Regarding education, policymakers can enhance ANC attendance by prioritizing maternal education programs that emphasize the significance of ANC for maternal and infant health. Integrating reproductive health education into school curricula can build awareness from an early age. Additionally, offering maternal health classes or informational sessions through community health workers can ensure access to ANC information for women with limited formal education.

Additionally, partner-inclusive ANC workshops or classes can help husbands understand the value of ANC, empowering them to support their spouses actively. Implementing policies in these areas can make ANC more accessible and practical for diverse groups, thereby reducing maternal and neonatal health disparities and promoting healthier families overall. However, this study highlights the societal importance of evidence-based policies aimed at preventing unintended pregnancies. Improved policy decisions in this domain could yield far-reaching benefits, including (i) enhanced public health outcomes by reducing maternal and neonatal complications linked to unplanned pregnancies; (ii) strengthened social welfare through better resource allocation for family planning, education, and childcare support; and (iii) positive economic ripple effects, such as increased workforce participation and reduced public expenditures on avoidable healthcare and social services. By addressing systemic barriers to ANC utilization — such as inequitable access to contraception and culturally insensitive care — this research aligns with global efforts to advance reproductive autonomy, gender equity, and the Sustainable Development Goals (e.g., SDG 3.7, 5.6). Although this analysis is based on the 2022 BDHS data, its findings have broader implications regarding reproductive health policies in other countries, such as Ethiopia, andglobally. In practical terms, these insights can guide governments, NGOs, and development partners to design and implement more effective strategies for preventing unintended pregnancies and improving reproductive health outcomes both in Bangladesh and similar contexts worldwide.

## 5. Limitations and future research

This study has several limitations that should be considered. First, the BDHS data used in this study rely heavily on self-reported, such as the number of ANC visits and other explanatory variables. Such self-reporting is susceptible to recall bias and social desirability bias, which may lead to inaccuracies in the data and affect model performance. Second, the cross-sectional design of the BDHS dataset limits our ability to draw causal inferences. While the machine learning models can identify associations between predictors and ANC utilization, they cannot establish the directionality or temporality of these relationships. Third, class imbalance analysis through sampling techniques such as SMOTE or class weighting, which may affect the predictive accuracy of the models, has not been applied. Fourth, the results might have limited generalizability. The data and model were developed specifically within the Bangladeshi context, and their applicability to other settings with different health systems, cultural norms, or socioeconomic structures remains uncertain. To address these limitations, future research should explore the use of longitudinal or panel data that would allow for the investigation of causal pathways and temporal dynamics in ANC utilization. Additionally, incorporating behavioral and service-quality-related variables—such as women’s health beliefs, attitudes, and perceptions of healthcare access—could improve model accuracy and provide a more comprehensive understanding of the predictors of ANC use. We will explore the application of class balancing techniques, such as SMOTE or class weighting, to address class imbalance. Finally, validating these models across different countries and regions would help determine the external validity and transferability of the findings to broader global health contexts.

## 6. Conclusion

These findings indicate that the machine learning–based RGmodel is highly effective in predicting ANC visits among women in Bangladesh. The model identified several influential predictors of ANC utilization, including age, wealth index, region, husband’s education, women’s education, and place of residence. These predictors reflect both individual and household-level socioeconomic conditions that significantly influence access to and use of ANC and broader maternal healthcare services. Based on the identified predictors, the study recommends expanding access to contraceptive methods among high-risk groups, integrating reproductive health education into community outreach programs, and prioritizing interventions for women with lower educational attainment or socioeconomic disadvantage. These actionable suggestions aim to support policymakers and public health professionals in designing targeted strategies to improve ANC utilization and reproductive health outcomes. Ultimately, implementing these interventions can make a meaningful contribution to achieving Sustainable Development Goal 3 (SDG-3), which aims to ensure healthy lives and promote well-being for all at all ages, particularly by reducing maternal and child mortality.
